# Incidence and disease spectrum of inherited metabolic diseases screened by tandem mass spectrometry in Huai'an from 2018 to 2024

**DOI:** 10.3389/fped.2025.1629840

**Published:** 2025-11-21

**Authors:** Ming-ming Ou, Chun-tao Sun, Li Zhang, Ling-li Kong, Lin-xin Zhang, Jun Zheng, Pei-ying Zhang, Yu-mei Wang, Xiao-fei Lin, Xin Yang

**Affiliations:** 1 Neonatal Disease Screening Center, Huai'an Maternal and Child Health Care Hospital Affiliated to Yangzhou University, Huai'an, China; 2 Neonatal Disease Screening Center, The Huai'an Maternity and Child Clinical College of Xuzhou Medical University, Huai'an, China; 3 Department of Child Health, Huai'an Maternal and Child Health Care Hospital Affiliated to Yangzhou University, Huai'an, China; 4 Department of Child Health, The Huai'an Maternity and Child Clinical College of Xuzhou Medical University, Huai'an, China; 5 Department of Pediatrics, Huai'an Maternal and Child Health Care Hospital Affiliated to Yangzhou University, Huai'an, China; 6 Department of Pediatrics, The Huai'an Maternity and Child Clinical College of Xuzhou Medical University, Huai'an, China

**Keywords:** tandem mass spectrometry, inherited metabolic disorders, next generation sequencing, NGS, pathogenic or likely pathogenic variant, genetic variant

## Abstract

**Objectives:**

This study aimed to determine the incidence and disease spectrum of inherited metabolic diseases (IMDs) among newborns in Huai'an City, China.

**Methods:**

Expanded newborn screening for IMDs using tandem mass spectrometry (MS/MS) enables the simultaneous analysis of more than 40 metabolites and the identification of approximately 50 types of IMDs. Next-generation sequencing (NGS), targeting hundreds of IMD-associated genes, was subsequently performed for genetic analysis of patients identified through screening. Between June 2018 and December 2024, in total, 161,966 newborns in Huai'an were screened using MS/MS. Ultimately, 57 patients were diagnosed with IMDs based on plasma amino acid and acylcarnitine profiling, urinary organic acid analysis, and molecular genetic testing, performed via NGS. Data were analyzed using descriptive statistics.

**Results:**

Fifty-seven cases of IMDs were diagnosed, corresponding to an overall incidence rate of 1 in 2,842. Among these, 28 cases involved amino acid metabolism disorders (1 in 5,785), 17 cases of organic acid metabolism disorders (1 in 9,527), and 12 cases of fatty acid oxidation disorders (1 in 13,497). The three most common IMDs were phenylalanine hydroxylase deficiency (1 in 8,098), primary carnitine deficiency (1 in 23,138), and methylmalonic acidemia (1 in 32,393). Genetic testing revealed variants in all 57 patients, with 75 variants identified across 17 IMD-associated genes. Recurrent variants were observed in five IMDs, including PAH gene variants c.728G>A, c.611A>G, and c.721C>T for phenylketonuria, PAH c.158G>A, c.721C>T, and c.728G>A for mild hyperphenylalaninemia, SLC22A5 c.1400C>G for primary carnitine deficiency, MMACHC c.609G>A, c.567dup and c.482G>A for methylmalonic acidemia, ACADS c.1055C>T, and c.1130C>T for short-chain acyl-CoA dehydrogenase deficiency, and ACADSB c.923G>A for 2-methylbutyrylglycinuria. All these recurrent variants were reported as pathogenic or likely pathogenic, except PAH c.158G>A, which was classified as a variant of uncertain significance.

**Conclusion:**

The majority of IMD patients in Huai'an carried pathogenic or potentially pathogenic variants identified through expanded newborn screening. Although MS/MS newborn screening followed by NGS confirmation cannot prevent the occurrence of IMDs, timely diagnosis combined with appropriate treatment and management can effectively prevent morbidity, reduce mortality, and support long-term symptom control. Overall, this study demonstrates that MS/MS-based newborn screening combined with molecular diagnosis was highly effective for the study early detection and management of IMDs in the Huai'an population.

## 1 Introduction

Inherited metabolic diseases (IMDs) constitute a large group of disorders in which a single gene defect results in a metabolic block, leading to biochemical abnormalities that may manifest at birth and/or later in life. IMDs are classified as single-gene genetic diseases, most of which follow an autosomal recessive pattern of inheritance. The majority of children with IMDs present with non-specific clinical manifestations at birth and may remain undiagnosed unless neonatal screening is conducted. Without prompt diagnosis and treatment, IMDs can impair the function of multiple organs and systems, cause progressive and irreversible damage to the nervous system, and lead to developmental delay or even death. These outcomes impose a considerable burden on affected families and society. Early diagnosis and timely intervention for neonates identified with IMDs through screening can prevent severe clinical consequences, such as mild to severe irreversible intellectual disability, lifelong disability, physical handicaps, coma, and early death (1).

Newborn screening (NBS) for IMDs represents a highly successful public-health initiative aimed at detecting life-threatening or long-term health conditions to reduce morbidity and mortality (2). Tandem mass spectrometry (MS/MS) serves as a cornerstone technology in NBS programs, enabling the rapid detection of numerous metabolites in dried blood spots (DBSs). The simultaneous quantification of amino acids and acylcarnitines allows identification of approximately 40–50 IMDs within a few days after birth (3). Expanded NBS using MS/MS has been widely adopted worldwide due to its advantages, including rapid and convenient analysis, significantly increased detection rates of IMDs (4), early diagnosis, prevention of premature death, and cost-effectiveness (5, 6).

With the advent of target capture and next-generation sequencing (NGS), it has become possible to sequence large panels of disease-related genes simultaneously, making NGS the preferred approach for identifying the genetic etiology in newborns with abnormal MS/MS screening results for IMDs. Since 2018, Huai'an City in China has implemented newborn screening for IMDs using tandem mass spectrometry, achieving a screening rate exceeding 98%. This study aimed to determine the incidence, disease spectrum, and gene spectrum of IMDs among newborns in Huai'an City, China.

## 2 Materials and methods

### 2.1 Subjects

**Figure 1:**
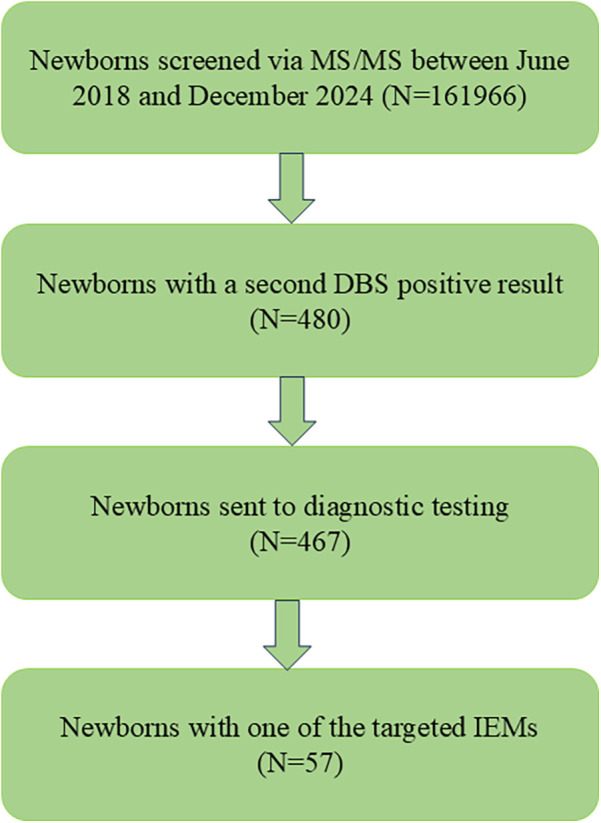
Flowchart of expanded newborn screening for IMDs and diagnosis of patients.

In total, 210,395 newborns were born in Huai'an between June 2018 and December 2024. Informed and written consent was obtained from the parents of 161,966 newborns, who were subsequently enrolled in the MS/MS-based NBS program. In total, 48,429 parents declined participation in the MS/MS NBS. Ultimately, 57 patients were diagnosed with IMDs through NGS. The screening protocol used in this study was consistent with that used by other newborn screening centers in China (Figure 1). The protocol was reviewed and approved by the Ethics Committee of Huai'an Maternal and Child Health Care Hospital, affiliated with Yangzhou University (Review No. 2022077).

### 2.2 Expanded newborn screening

Heel-prick blood samples were collected from the newborns, and 11 amino acids, 30 acylcarnitines, free carnitine, and succinylacetone were analyzed as biomarkers for 27 IMDs using a non-derivatized MS/MS method at the Huai'an Newborn Disease Screening Center. Samples were collected at between 48 h and 20 days after birth (median: 4 days). Screening assays were performed using a commercial kit (PerkinElmer, USA) and a Waters HPLC-MS/MS system (TQD, Waters, USA). For each patient, a 3.2-mm DBS punch was taken using a Puncher 9 automatic drilling instrument (PerkinElmer, Finland) and placed in a 96-well U-bottom plate. Then, 100 µl of extraction solution containing internal standards was added to each well. After incubation at 45 °C for 45 min, 90 µl of the extract was transferred to a V-bottom plate and left at room temperature for 2 h. Subsequently, 25 µl of the extract was injected into the MS/MS instrument for metabolite analysis. Three levels of internal quality control—blank, low, and high were included in each batch to ensure analytical reliability.

### 2.3 Positive results for IMDs

The screening panel encompassed 27 types of IMDs. Each IMD was defined by two or more diagnostic indicators, including metabolites and metabolite ratios, with specific cut-off values. DBS results that met the positive criteria for IMDs were classified as suspected positive cases.

### 2.4 Genetic analysis

#### 2.4.1 DNA extraction

In total, 5 ml of peripheral whole blood was collected from each subject in EDTA-containing anticoagulant tubes. Genomic DNA was extracted from 2 ml of peripheral blood using a Qiagen Blood DNA Mini Kit (Qiagen®, Hilden, Germany). DNA concentration was measured, and the extracted samples were stored at −20 °C. The remaining whole blood samples were stored at −80 °C for future use.

#### 2.4.2 High throughput sequencing

High-throughput sequencing was conducted for all patients clinically diagnosed with IMDs using an expanded IMD gene panel (Genuine Diagnostic, Hangzhou, China) that included 346 genes related to IMDs. Target regions were captured using multiple probe hybridization, and the resulting capture products were purified with Agencourt AMPure XP beads (Beckman Coulter). The purified DNA was processed using a TruePrep™ DNA Library Prep Kit V2 for Illumina (Vazyme) and indexed with a TruePrep™ Index Kit V2 for Illumina (Vazyme). Library quality was assessed using a Qubit fluorometer and an Agilent 2100 bioanalyzer (High Sensitivity DNA Kit, Agilent Technologies). Sequencing libraries were quantified using an Illumina DNA Standards and Prime Premix Kit (Kapa Biosystems) and then subjected to massively parallel sequencing on the DNBSEQ-T7 platform. Paired-end reads were quality-trimmed using the Trimmomaticprogram and aligned to the human reference genome (UCSC Genome). Single-nucleotide polymorphisms and insertions/deletions were identified using the SAMtoolsprogram.

#### 2.4.3 Sanger sequencing

All variants identified through high-throughput sequencing were confirmed by Sanger sequencing using specific primers. Polymerase chain reaction (PCR) amplification was performed using aTaKaRa LA PCR™ Kit ver. 2.1 (TaKaRa). PCR products were purified from agarose gels using aNucleoSpin® Gel and PCR Clean-up Kit (MACHEREY-NAGEL). Purified PCR products were diluted to a final concentration of 10 ng/µl and subjected to sequencing PCR using aBigDye® Terminator v3.1 Cycle Sequencing Kit (Applied Biosystems). Each reaction well received 10 µl of Hi-Di™ Formamide (Applied Biosystems). The samples were denatured at 95 °C for 5 min, cooled, and transferred to 96-well plates. Sequencing was performed using an ABI 3500XL Genetic Analyzer (Applied Biosystems).

### 2.5 Statistical analysis

Statistical analyses were conducted using SPSS software version 26.0 (IBM Corp., Armonk, NY, USA). Descriptive statistics were applied for count data.

## 3 Results

### 3.1 Screening of IMDs with MS/MS

In total, 161,966 newborns were screened using the expanded NBS program in Huai'an (Figure 1). Following the initial screening and subsequent repeat testing, 480 newborns with a second positive result were classified as “suspected positive”. Of these, 467 underwent NGS diagnostic testing, while the remaining 13 did not proceed with NGS. The biochemical indicators in these 13 newborns were only mildly abnormal, and their parents declined further genetic testing. During follow-up, the previously abnormal biochemical indicators in these cases returned to normal. Ultimately, 57 patients were diagnosed with IMDs based on MS/MS testing, urinary organic acid analysis, and molecular genetic testing using NGS.

### 3.2 Distribution of disease spectrum in children with IMDs

**Figure 2:**
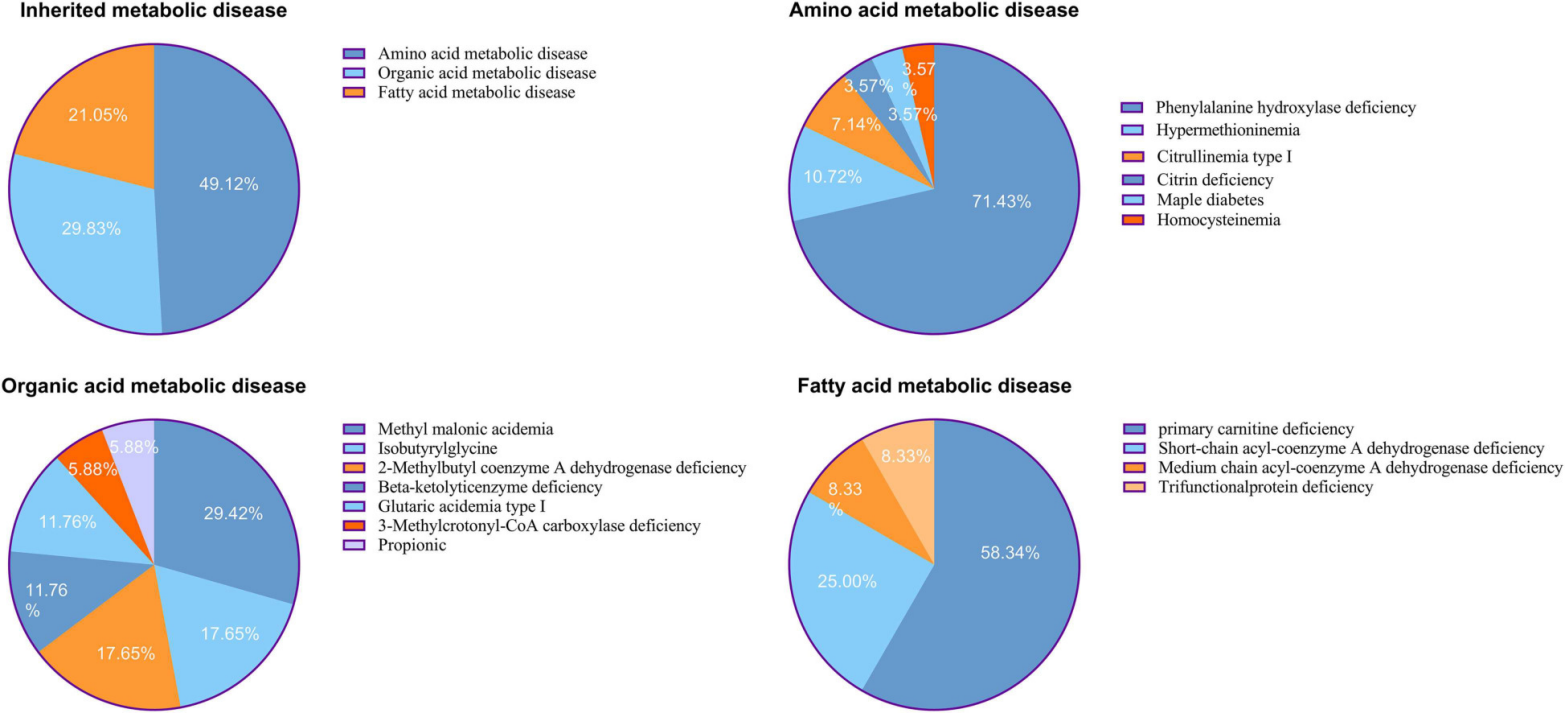
**(A)** Distributions of inherited metabolic disease. **(B)** Distributions of amino acid metabolic disease. **(C)** Distributions of organic acid metabolic disease. **(D)** Distributions of fatty acid metabolic disease.

In total, 57 cases of IMDs were confirmed, corresponding to an overall incidence rate of 1 in 2,842 live births. Among these, 28 cases were amino acid metabolism disorders (1 in 5,785), 17 were organic acid metabolism disorders (1 in 9,527), and 12 were fatty acid oxidation disorders (1 in 13,497). The three most common IMDs by incidence were phenylalanine hydroxylase deficiency (1 in 8,098), primary carnitine deficiency (PCD; 1 in 23,138), and methylmalonic acidemia (MMA; 1 in 32,393) (Table 1, Figures 2A–D).

**Table 1:** Disease spectrum of 57 children with IMDs in 161,966 newborns.

Disease name	Number of confirmed cases	Morbidity	Constituent ratio (%)	Constituent ratio (%)
**Amino acid metabolic disease**	28	1:5,785	49.12%	
Phenylalanine hydroxylase deficiency	20	1:8,089		71.43%
Hypermethioninemia	3	1:53,989		10.72%
Homocysteinemia	1	1:161,966		3.57%
Citrullinemia type 1	2	1:80,983		7.14%
Citrin deficiency	1	1:161,966		3.57%
Maple syrupurine disease	1	1:161,966		3.57%
**Organic acid metabolic disease**	17	1:9,527	29.83%	
Methylmalonic acidemia	5	1:32,393		29.42%
Isobutyrylglycine uria	3	1:53,989		17.65%
Glutaric acidemia type I	2	1:80,983		11.76%
2-Methylbutyl coenzyme A dehydrogenase deficiency	3	1:53,989		17.65%
Propionic acidemia	1	1:161,966		5.88%
3-methylcrotonyl-CoA carboxylase deficiency	1	1:161,966		5.88%
Beta-ketolytic enzyme deficiency	2	1:80,983		11.76%
**Fatty acid metabolic disease**	12	1:13,497	21.05%	
primary carnitine deficiency	7	1: 23,138		58.34%
Short-chain acyl-coenzyme A dehydrogenase deficiency	3	1:53,989		25.00%
Medium chain acyl-coenzyme A dehydrogenase deficiency	1	1:161,966		8.33%
Trifunctional protein deficiency	1	1:161,966		8.33%

### 3.3 Results of MS/MS in 57 children with IMDs

The results from the primary MS/MS screening and secondary recall testing in 57 children with IMDs revealed characteristic abnormalities in disease-related indices. The corresponding metabolite ratios and indicative values are presented in Table 2.

**Table 2:** Results of MS/MS in children with IMDs (μmol/L).

Disease name	Number	MS/MS characteristic anomaly index	Reference value	Readouts in primary screening [X(min–max)]	Readout in secondary screening and reviewing [X(min–max)]
Phenylalanine hydroxylase deficiency	20	PHE	25.00–90.00	518.78 (109.06–1870.4)	838.91 (112.57–2695.8)
		PHE/TYR	0.20–1.56	9.07 (1.42–39.74)	11.24 (1.17–40.99)
Hypermethioninemia	3	MET	7.50–40.00	59.38 (32.58–81.88)	108.92 (37.76–239.36)
		MET/ PHE	0.15–0.77	1.29 (1.02–1.81)	3.24 (1.19–7.25)
Homocysteinemia	1	MET	7.50–40	4.65	5.18
		MET/PHE	0.15–0.77	0.13	0.19
Citrullinemia type 1	2	CIT	6.20–30.00	112.48 (88.81–136.15)	138.74 (105.95–171.53)
		CIT/PHE	0.10–0.72	2.18 (1.62–2.73)	2.75 (2.08–3.41)
Citrin deficiency	1	CIT	6.20–30.00	216.38	60.76
		CIT/PHE	0.10–0.72	1.25	1.72
Maple syrupurine disease	1	LEU+ILE+PRO-OH	88.00–276.00	1976.58	2935.24
		Leu+ILE+PRO-OH/PHE	1.61–5.54	52.35	90.60
		VAL	65.00–233.00	566.21	328.17
		VAL/PHE	1.31–4.63	15.00	10.13
Methylmalonic acidemia	5	C3	0.40–3.90	8.98 (4.16–15.41)	6.46 (2.86–13.04)
		C3/C2	0.04–0.24	0.62 (0.32–1.08)	0.82 (0.39–1.15)
		C3/C0	0.02–0.18	0.40 (0.23–0.78)	0.35 (0.14–0.74)
Isobutyrylglycine uria	3	C4	0.10–0.48	1.28 (1.15–1.52)	1.82 (1.42–2.14)
		C4/C3	0.05–0.40	1.85 (1.58–2.21)	2.04 (1.47–2.75)
		C4/C8	1.21–10.67	26.83 (19.5–38.00)	44.17 (31.67–53.50)
Glutaric acidemia type Ⅰ	2	C5DC+C6OH	0.04–0.20	2.47 (2.31–2.62)	1.38 (0.98–1.78)
		C5DC+C6OH/C8	0.60–4.50	55.90 (46.20–65.60)	69.00 (49.00–89.00)
		C5DC+C6OH/C4DC+C5OH	0.18–1.10	9.86 (9.24–10.28)	6.10 (4.46–7.74)
2-Methylbutyl coenzyme A dehydrogenase deficiency	3	C5	0.04–0.27	0.48 (0.44–0.51)	0.83 (0.72–0.94)
		C5/C3	0.02–0.32	0.34 (0.16–0.44)	1.19 (0.78–1.45)
Propioninemia^a^	1	C3	0.40–3.90	14.39	/
		C3/C2	0.04–0.24	1.31	/
		C3/C0	0.02–0.18	1.24	/
3-methylcrotonyl-CoA carboxylase deficiency	1	C4DC+C5OH	0.08–0.48	9.43	10.24
		C4DC+C5OH/C0	0.00–0.03	2.97	4.15
		C4DC+C5OH/C8	0.90–13.00	471.50	1024.00
Beta-ketolytic enzyme deficiency	2	C4DC+C5OH	0.08–0.48	1.30 (1.10–1.49)	1.18 (1.02–1.34)
		C3DC+C4OH	0.02–0.32	0.84 (0.66–1.02)	0.31 (0.25–0.37)
		C5:1	0.00–0.02	0.37 (0.36–0.38)	0.29 (0.24–0.34)
Primary carnitine deficiency	7	C0	9.00–49.00	6.66 (3.58–8.91)	5.24 (1.66–7.41)
Short chain acyl-coenzyme A dehydrogenase deficiency	3	C4	0.10–0.48	1.33 (0.80–2.13)	1.25 (1.10–1.52)
		C4/C2	0.00–0.05	0.10 (0.06–0.15)	0.16 (0.13–0.20)
		C4/C3	0.05–0.40	1.00 (0.75–1.14)	1.49 (1.39–1.59)
Medium chain acyl-coenzyme A dehydrogenase deficiency	1	C8	0.01–0.15	0.45	0.33
		C8/C2	0.00–0.01	0.04	0.04
		C8/C10	0.40–1.50	3.00	4.13
Trifunctionalprotein deficiency	1	C12	0.01–0.22	0.05	0.46
		C14	0.03–0.36	0.07	0.55
		C14-OH	0–0.03	0.03	0.13
		C16	0.33–5.54	0.40	1.33
		C16-OH	0–0.04	0.10	0.22
		C18	0.13–1.47	0.38	0.46
		C18-OH	0–0.03	0.10	0.54

All ratios have no units.

a No data from secondary screening is available as propioninemia is a fatal disease and all subjects died before secondary screening.

### 3.4 Variants in IMD patients identified by expanded newborn screening

Genetic analysis identified pathogenic or likely pathogenic variants in all 57 patients diagnosed with IMDs. In total, 75 variants were detected across 17 IMD-associated genes. Several recurrent variants were observed among 5 IMD types, including PAH gene variants c.728G>A, c.611A>G, and c.721C>T in phenylketonuria; PAH gene variants c.158G>A, c.721C>T, and c.728G>A in mild hyperphenylalaninemia; SLC22A5 gene variant c.1400C>Gin PCD; MMACHC gene variants c.609G>A, c.567dup, and c.482G>A in MMA; ACADS gene variants c.1055C>T and c.1130C>Tin short-chain acyl-CoA dehydrogenase deficiency (SCADD); and ACADSB gene variant c.923G>A in 2-methylbutyryl-CoA dehydrogenase deficiency (SBCADD). All of these recurrent variants have previously been reported as pathogenic or likely pathogenic, except for the PAH gene variant c.158G>A, which remains of uncertain significance (Table 3).

**Table 3:** Mutations in 57 patients with IEMs identified by expanded newborn screening.

Conditions (OMIM number)	Gene (OMIM number)	Mutation alleles number	Nucleotide variant	Amino acid variant	Pathogenic	RF%	Cases	Accounting for patients (%)
Phenylketonuria (261,600)	PAH (612,349)	22					11	
		7	c.728G>A	p.Arg243Gln	P	31.82%	6	54.55%
		4	c.611A>G	p.Tyr204Cys	P	18.18%	3	27.27%
		3	c.721C>T	p.Arg241Cys	P	13.64%	3	27.27%
		1	c.598dup	p.Thr200AsnfsTer6	P	4.55%	1	9.09%
		1	c.1068C>A	p.Tyr356Ter	P	4.55%	1	9.09%
		1	c.331C>T	p.Arg111Ter	P	4.55%	1	9.09%
		1	c.509+1G>A	–	P	4.55%	1	9.09%
		1	c.1045T>G	p.Ser349Ala	P	4.55%	1	9.09%
		1	c.699C>G	p.Phe233Leu	P	4.55%	1	9.09%
		1	c.1301C>A	p.Ala434Ser	P	4.55%	1	9.09%
		1	c.194T>C	p.Ile65Thr	P	4.55%	1	9.09%
Mild hyperphenylalaninemia (261,600)	PAH (612,349)	18					9	
		4	c.158G>A	p.Arg53His	VUS	22.22%	4	44.44%
		4	c.721C>T	p.Arg241Cys	P	22.22%	2	22.22%
		2	c.728G>A	p.Arg243Gln	P	11.11%	2	22.22%
		1	c.929C>T	p.Ser310Phe	P	5.56%	1	11.11%
		1	c.301G>A	p.Asp101Asn	LP	5.56%	1	11.11%
		1	c.1256A>G	p.Gln419Arg	P	5.56%	1	11.11%
		1	c.611A>G	p.Tyr204Cys	P	5.56%	1	11.11%
		1	c.460T>C	p.Tyr154His	P	5.56%	1	11.11%
		1	c.827T>A	p.Met276Lys	LP	5.56%	1	11.11%
		1	c.331C>T	p.Arg111Ter	P	5.56%	1	11.11%
		1	c.1031G>A	p.Gly344Asp	LP	5.56%	1	11.11%
Primary carnitine deficiency (212,140)	SLC22A5 (603,377)	14					7	
		9	c.1400C>G	p.Ser467Cys	P	64.29%	6	85.71%
		1	c.506G>A	p.Arg169Gln	P	7.14%	1	14.29%
		1	c.498-2A>T	–	LP	7.14%	1	14.29%
		1	c.338G>A	p.Cys113Tyr	P	7.14%	1	14.29%
		1	c.760C>T	p.Arg254Ter	P	7.14%	1	14.29%
		1	c.51C>G	p.Phe17Leu	P	7.14%	1	14.29%
Methylmalonic aciduria and homocystinuria, cblC type (277,400)	MMACHC (609,831)	10					5	
		3	c.609G>A	p.Trp203Ter	P	30.00%	2	40.00%
		2	c.567dup	p.Ile190TyrfsTer13	LP	20.00%	2	40.00%
		2	c.482G>A	p.Arg161Gln	P	20.00%	2	40.00%
		1	c.599G>A	p.Trp200Ter	P	10.00%	1	20.00%
		1	c.80A>G	p.Gln27Arg	P	10.00%	1	20.00%
		1	exon1 del	–	LP	10.00%	1	20.00%
Isobutyryl coa dehydrogenase deficiency (611,283)	ACAD8 (604,773)	6					3	
		1	c.1000C>T	p.Arg334Cys	LP	16.67%	1	33.33%
		1	g.134122267_134123466del	–	US	16.67%	1	33.33%
		1	c.286G>A	p.Gly96Ser	P	16.67%	1	33.33%
		1	c.526G>A	p.Ala176Thr	US	16.67%	1	33.33%
		1	c.G236G>A	p.Arg79Gln	US	16.67%	1	33.33%
		1	c.568-3C>G	–	US	16.67%	1	33.33%
Short-chain acyl-Coa dehydrogenase deficiency (201,470)	ACADS (606,885)	6					3	
		2	c.1055C>T	p.Ala352Val	LP	33.33%	2	66.67%
		2	c.1130C>T	p.Pro377Leu	LP	33.33%	1	33.33%
		1	c.881C>T	p.Ala294Val	US	16.67%	1	33.33%
		1	c.164C>T	p.Pro55Leu	P	16.67%	1	33.33%
2-Methylbutyrylglycinuria (610,006)	ACADSB (600,301)	6					3	
		2	c.923G>A	p.Cys308Tyr	LP	33.33%	1	33.33%
		1	c.1172del	p.Gly391GlufsTer2	LP	16.67%	1	33.33%
		1	c.848A>G	p.Tyr283Cys	LP	16.67%	1	33.33%
		1	c.655G>A	p.Val219Met	LP	16.67%	1	33.33%
		1	c.461G>A	p.Gly154Glu	VUS	16.67%	1	33.33%
Glutaricaciduria, type I (231,670)	GCDH (608,801)	4					2	
		1	c.1091C>T	p.Pro364Leu	US	25.00%	1	50.00%
		1	c.769C>T	p.Gly243Glu	LP	25.00%	1	50.00%
		1	c.1199T>C	–	LP	25.00%	1	50.00%
		1	c.1205G>A	p.Arg402Gln	P	25.00%	1	50.00%
Hypermethioninemia (250,850)	MAT1A (610,550)	4					3	
		1	c.895C>T	p.Arg299Cys	P	25.00%	1	33.33%
		1	c.754A>T	p.Ile252Phe	LP	25.00%	1	33.33%
		1	c.776C>T	p.Ala259Val	LP	25.00%	1	33.33%
		1	c.746G>A	p.Arg249Gln	LP	25.00%	1	33.33%
Beta-ketolytic enzyme deficiency(203,750)	ACAT1 (607,809)	4					2	
		1	c.121-3C>G	–	US	25.00%	1	50.00%
		1	c.275G>A	p.Gly92Ser	LP	25.00%	1	50.00%
		1	c.1124A>G	p.Asn375Ser	P	25.00%	1	50.00%
		1	c.631C>A	p.Gln211Lys	VUS	25.00%	1	50.00%
Citrullinemia type 1 (215,700)	ASS1 (603,470)	4					2	
		1	c.349G>A	p.Gly117Ser	P	25.00%	1	50.00%
		1	c.805G>A	p.Val269Met	LP	25.00%	1	50.00%
		1	c.919C>T	p.Arg307Cys	LP	25.00%	1	50.00%
		1	c.1069C>G	p.Gln357Glu	VUS	25.00%	1	50.00%
Citrin deficiency (605,814)	SLC25A13 (603,859)	2					1	
		1	c.1048G>A	p.Asp350Asn	P	50.00%	1	100.00%
		1	c.852_855del	p.M285Pfs*2	P	50.00%	1	100.00%
Hyperhomocysteinemia (236,250)	MTHFR (607,093)	2					1	
		2	c.1316T>C	p.Leu439Pro	LP	100.00%	1	100.00%
Maple syrup urine disease, type Ib (620,698)	BCKDHB (248,611)	2					1	
		1	c.93_103dup	p.Phe35TrpfsTer41	LP	50.00%	1	100.00%
		1	c.673_675del	p.Leu226del	US	50.00%	1	100.00%
Propionicacidemia (606,054)	PCCB (232,050)	2					1	
		1	c.838dup	p.Leu280ProfsTer11	P	50.00%	1	100.00%
		1	c.1228C>T	p.Arg410Trp	P	50.00%	1	100.00%
3-Methylcrotonyl-Coa carboxylase deficiency type 2 (210,210)	MCCC2 (609,014)	2					1	
		2	c.728G>A	p.Gly243Glu	US	100.00%	1	100.00%
Medium-chain acyl-Coa dehydrogenase deficiency (201,450)	ACADM (607,008)	2					1	
		1	c.449_452del	p.Thr150ArgfsTer4	LP	50.00%	1	100.00%
		1	c.860G>A	p.Gly287Asp	US	50.00%	1	100.00%
Trifunctional protein deficiency (620,300)	HADHB (143,450)	2					1	
		1	c.209+1G>A	–	P	50.00%	1	100.00%
		1	c.520C>T	p.Arg174Cys	LP	50.00%	1	100.00%

## 4 Discussion

There are many types of IMDs, and the total number of identified disorders is estimated to exceed several thousand. Although the incidence of any single IMD is generally low, ranging from several per 10,000 to several per 100,000 live births, the cumulative incidence of all IMDs is considerably higher. Since the early 1990s, MS/MS has been widely implemented in neonatal screening programs for IMDs due to its relatively low cost, high sensitivity (99%), and high specificity (>99.8%) (7). The majority of IMDs detected through MS/MS are disorders of organic acid, amino acid, or fatty acid metabolism. Reported IMD types and their incidences vary substantially among countries and regions, reflecting differences in screening panels, population genetics, and healthcare systems.

As summarized in Table 4, expanded newborn screening programs worldwide demonstrate diverse detection rates. For example, approximately 40 types of IMDs are screened in Italy, with an incidence of about 1 in 6,041 (4). In Spain, 13 IMDs are screened with an incidence of approximately 1 in 2,577 (8). Germany, Japan, and Korea screen for 24, 18, and 24 IMDs, respectively, with corresponding incidences of 1/2,200, 1/8,557, and 1/13,205 (9). In China, 38 IMDs are screened by MS/MS across multiple neonatal screening centers, with an overall incidence of about 1 in 1,410 (10). In the present study, in total 161,966 newborns were screened in Huai'an, and 57 IMD cases were confirmed by NGS, corresponding to an incidence of 1/2,842. The three most common IMDs were phenylalanine hydroxylase deficiency (1/8,098), PCD (1/23,138), and MMA (1/32,393), consistent with previously reported data from other regions of China (10). All 57 affected children exhibited biochemical abnormalities of varying degrees that were successfully detected by MS/MS, confirming its reliability and critical role in early IMD screening and diagnosis.

**Table 4:** Comparison of expanded newborn screening detection incidences of inherited metabolic diseases per country.

Author	Publication year	Country	Types of IMDs	Sample size (*n*)	Incidence rates of IMDs
Messina M, et al. (4)	2018	Italy^a^	40	60,408	1/6,041
Cambra Conejero A, et al. (8)	2020	Spain^b^	13	592,822	1/2,577
Shibata N, et al. (9)	2018	Germany^c^	24	7,510,000	1/2,200
Shibata N, et al. (9)	2018	Japan^d^	18	3,360,000	1/8,557
Shibata N, et al. (9)	2018	Korea^e^	24	3,440,000	1/13,205
Tang C, et al. (10)	2024	China^f^	38	29,601	1/1,410

a Data from Italy are from 2011 to 2017.

b Data from Spain are from 2011 to 2019.

c Data from Germany are from 2002 to 2015.

d Data from Japan are from 1997 to 2015.

e Data from Korea are from 2000 to 2015.

f Data from China are from February 2021 to December 2021.

Hyperphenylalaninemia is the most prevalent amino acid metabolism disorder, resulting from a deficiency of either phenylalanine hydroxylase (PAH) or its cofactor, tetrahydrobiopterin (BH4). The deficiency leads to elevated phenylalanine concentrations in the blood, which can cause neurotoxicity and irreversible brain damage if left untreated. To date, more than 800 PAH gene variants have been identified in patients with PAH deficiency (11), with several recurrent variants reported in distinct populations. The most frequent variants include c.168+5G>C in Western Iranians (12), IVS10-11G>A in Iranians (13, 14) and Spaniards (15), c.1238G>C in Japanese (16), c.1162G>A in Brazilians (17), c.728G>A in Chinese (11, 18, 19), c.1068C>A and c.728G>A in South Koreans (20), c.1222C>T in Australians (21), and c.782G>A in Syrians (22).

In the Huai'an cohort, all 20 patients diagnosed with PAH deficiency harbored variants in the PAH gene. The detected variant included c.728G>A, c.611A>G, c.721C>T, c.598dup, c.1068C>A, c.331C>T, c.509+1G>A, c.1045T>G, c.699C>G, c.1301C>A, c.194T>C, c.158G>A, c.929C>T, c.301G>A, c.1256A>G, c.460T>C, c.827T>A, and c.1031G>A. Consistent with findings from other Chinese populations, the most frequent variant in this study was c.728G>A, accounting for 22.5% of alleles, followed by c.721C>T (17.5%) and c.611A>G (12.5%).

PCD was the second most common IMD detected in this study. PCD is caused by defects in the carnitine transporter protein encoded by the SLC22A5 gene, leading to impaired fatty acid β-oxidation within cells. The reported incidence of PCD in China varies regionally, ranging from 1/26,777 to 1/8,938 (18, 23, 24), while the incidence in Huai'an was 1/23,138. More than 110 SLC22A5variants have been identified to date, with distinct recurrent variants observed across different ethnic groups (25). For example, c.844T>C is common among Caucasian patients (26–28), while c.1400C>Gis the predominant hotspot variant in Southeast Asian populations (29–31). In Chinese populations, recurrent variants are relatively consistent across regions. The c.1400C>G variant is the most frequent, with a relative frequency of 50%, accounting for 80% of PCD cases in Suzhou (22). Chen et al. reported c.760C>T (32.9%) and c.1400C>G (21.1%) as the most common variants (32). Similarly, in the Huai'an cohort, c.1400C>G was the predominant SLC22A5 variant, with a relative frequency of 64.29%, aligning with findings from most other studies.

MMA was identified as the third most prevalent IMD and the most common organic acid metabolic disorder among the Huai'an population. MMA can be classified according to the specific defect into two categories: cobalamin (cbl) metabolic defects and methylmalonyl-CoA mutase deficiency (MUT type). It is a genetically and phenotypically heterogeneous disorder characterized by diverse clinical manifestations and significant morbidity. Currently, isolated MMA is attributed to variants in five genes—MMADHC, MUT, MCEE, MMAA, and MMAB. Among Chinese populations, MUT gene variants are the most frequently observed pathogenic type, with more than 400 related variant sites cataloged in ClinVar. The cblC type of MMA is the most common form associated with concurrent homocysteinemia. Reported incidences of MMA are approximately 1/50,000 in Japan (33) and 1/250,000 in Germany (34). However, in mainland China, the incidence ranges from 1/3,920 to 1/26,000 (18, 35). The incidence of MMA in Huai'an, approximately 1/32,393, is evidently higher than that in Japan and Germany but lower than in Shandong, Henan, Beijing, Shanghai, and Suzhou.

In this study, five patients were diagnosed with MMA, and all were classified as the cblC type. These patients carried six MMACHC gene variants: c.609G>A, c.567dup, c.482G>A, c.599G>A, c.80A>G, and an exon 1 deletion. These variants accounted for 28.6% of the total detected genetic sites, a distribution consistent with findings from most Chinese studies (36–38). All children diagnosed with MMA in the present cohort exclusively exhibited the combined type, and no variants in other related genes were identified. Given the limited sample size, the precision of variant frequency representation in the affected population may be constrained. Nevertheless, the identification of recurrent MMACHC gene variants—c.609G>A, c.567dup, and c.482G>A—highlights their importance as potential hotspots for subsequent MMA gene screening, prenatal diagnosis, and genetic counseling.

SCADD is an autosomal recessive disorder of fatty acid oxidation caused by defects in the ACADS gene, leading to the accumulation of butyrylcarnitine (C4) and ethylmalonic acid in urine. The ACADS gene is located on chromosome 12q24.31, spans approximately 13 kb, contains 10 exons, and encodes a 412-amino-acid enzyme. In this study, three cases of SCADD were identified with ACADS gene variants at four sites: c.1055C>T, c.881C>T, c.1130C>T, and c.164C>T. Among these, c.1055C>T and c.1130C>T exhibited the highest variant frequencies, each accounting for 33.33% of the detected sites. According to the American College of Medical Genetics and Genomics (ACMG) variant classification standards, c.881C>T (p.Ala294Val) was deemed clinically insignificant. Variants c.511C>T and c.625G>A are prevalent in Euramerican and Jewish populations (39), whereas c.1031A>G, c.164C>T, and c.323G>A have been reported in Japan (40). Long-term follow-up studies of SCADD cases detected through newborn screening have shown most patients remain asymptomatic, underlying a rationale that SCADD should not be routinely screened in newborns (41, 42). SCADD has been labeled a “biochemical phenotype” rather than a clinically significant monogenic disorder (43). This remains a controversial issue regarding the clinical significance of SCADD and whether it should be included in newborn screening panels.

Isobutyryl-CoA dehydrogenase deficiency (IBDD) is a rare autosomal recessive metabolic disorder that results from defects in the ACAD8gene, which encodes an enzyme responsible for the catabolism of the essential amino acid valine. Disruption of this metabolic pathway leads to the accumulation of isobutyrylglycine, which is excreted in urine and detectable through organic acid analysis. Accordingly, the condition is often referred to as isobutyrylglycinuria. The ACAD8gene is located on chromosome 11q25, comprises 11 exons, and encodes a member of the acyl-CoA dehydrogenase family. In this study, three cases of isobutyrylglycinuria were diagnosed, all associated with ACAD8 gene variants. Five variants— c.1000C>T, c.286G>A, c.526G>A, c.236G>A, and c.568-3C>G—as well as one structural variant (g.UNK1134123466 deletion) were identified. Based on ACMG classification criteria, c.526G>A, c.236G>A, and c.568-3C>G were categorized as variants of uncertain clinical significance. Reports from both Chinese and international studies imply that most IBDD patients are asymptomatic, making it difficult to establish clear genotype-phenotype correlations.

The etiology of hyperhomocysteinemia (HHCY) is multifactorial and can be categorized into congenital and acquired causes. Congenital factors primarily involve genetic variants in key enzymes such as thioether-β-synthase, methionine synthase reductase, methylenetetrahydrofolate reductase (MTHFR), and methionine synthase. These genetic defects lead to deficiencies in cofactors required for homocysteine (HCY) metabolism, resulting in HCY accumulation and subsequent development of HHCY. In the present study, one case of homocysteinemia was identified, in which a homozygous variant of the MTHFR gene (c.1316T>C) was detected. This case was initially identified through MS/MS screening, which revealed methionine levels below the diagnostic threshold, and was subsequently confirmed through molecular diagnosis using NGS. These findings indicate that HHCY can be detected by MS/MS-based newborn screening, acknowledging that screening for low methionine without screening for elevated HCY wouldn't effectively screen for all genetic etiologies of HHCY.

Citrullinemia type I (CTLN1), also referred to as argininosuccinate synthase (ASS) deficiency, is caused by pathogenic variants in the ASS1gene, a key enzyme in the urea cycle. The disorder is characterized by hyperammonemia and elevated citrulline levels and follows an autosomal recessive inheritance pattern (44). The ASS1 gene is located on chromosome 9q34.11, spans approximately 56 kb, comprises 16 exons, and encodes a 412-amino-acid protein. Reported common variants include c.1085G>T and c.970G>A in Turkish patients, and c.1088G>A in German patients (45, 46). In East Asian populations, c.421-2A>G is the most frequently observed variant, whereas c.970G>A predominates in Korean patients (47, 48). Additionally, c.794G>A is the most common variant reported among Japanese CTLN1 patients (49). In the present study, two cases of CTLN1 were diagnosed, and four heterozygous ASS1 gene variants were detected: c.349G>A, c.805G>A, c.919C>T, and c.1069C>G.

Maple syrup urine disease (MSUD) is an autosomal recessive disorder affecting the catabolism of branched-chain amino acids. The condition derives its name from the characteristic sweet, “maple syrup–like” odor of the urine. MSUD results from a functional defect in the branched-chain *α*-keto acid dehydrogenase complex. The primary pathogenic genes implicated in this disorder include BCKDHA (34%), BCKDHB (29%), DLD, and DBT. In this study, we identified an MSUD case in which two heterozygous variants of the BCKDHB gene were detected: c.93_103dup and c.673_675del. According to the ACMG variant classification guidelines, the c.673_675del (p.Leu226del) variant is classified as having uncertain clinical significance, warranting further functional validation. It shows how NGS as a standalone first tier screen would be insufficient as VUS clinical correlation is only feasible because of the MS/MS NBS result.

3-Methylcrotonyl-CoA carboxylase deficiency (3-MCCD) is an autosomal recessive inborn error of leucine metabolism that exhibits considerable phenotypic variability. Most affected individuals remain asymptomatic unless secondary systemic carnitine deficiency develops. However, severe cases may present with leukodystrophy, developmental delay, hypoglycemia, metabolic acidosis, failure to thrive, lactic acidosis, and hyperammonemia (50). Deficiency of 3-MCCC, caused by pathogenic variants in either MCCC1 or MCCC2, leads to elevated levels of 3-hydroxyisovalerylcarnitine in the blood. The enzyme comprises *α* and β subunits encoded by the MCCC1 and MCCC2 genes, respectively. Previous studies have shown that MCCC2variants are more frequently reported worldwide, whereas MCCC1variants are predominant in Chinese populations (51). In the present study, one patient with 3-MCCD was diagnosed, carrying a homozygous MCCC2 gene variant (c.728G>A).

## 5 Conclusions

This study reports, for the first time, the results of newborn screening for IMDs and their epidemiological characteristics in Huai'an over the past 7 years. Among 161,966 newborns screened using MS/MS, 57 children were diagnosed with 17 different types of IMDs, yielding an overall incidence rate of 1 in 2,842. The three most prevalent disorders were phenylalanine hydroxylase deficiency, PCD, and MMA, findings that were consistent with the reported incidence patterns of these diseases in other regions of China.

Of the total 77 genetic variants identified, 49.35% (38/77) were classified as pathogenic, 32.47% (25/77) as likely pathogenic, and 18.18% (14/77) as variants of uncertain clinical significance, the latter requiring further functional validation. Several recurrent variants were identified across 10 IMDs, including PAH gene variants (c.728G>A, c.611A>G, and c.721C>T) for phenylketonuria; PAH gene variants (c.158G>A, c.721C>T, and c.728G>A) for mild hyperphenylalaninemia; SLC22A5gene variant (c.1400C>G) for PCD; MMACHC gene variants (c.609G>A, c.567dup, and c.482G>A) for MMA; ACADS gene variants (c.1055C>T and c.1130C>T) for SCADD; and ACADSB gene variant (c.923G>A) for SBCADD. All these recurrent variants have been previously reported as pathogenic or likely pathogenic, except for the PAH gene variant c.158G>A.

In conclusion, the majority of the IMD patients identified through expanded newborn screening in Huai'an carried pathogenic or potentially pathogenic variants. These findings underscore the clinical value of combining MS/MS-based screening with NGS for the accurate diagnosis and genetic confirmation of IMDs in newborns.

## Data Availability

The original contributions presented in the study are included in the article/Supplementary Material, further inquiries can be directed to the corresponding authors.
